# Prevalence of canine *Babesia* and *Ehrlichia* co-infection and the predictive value of haematology

**DOI:** 10.4102/ojvr.v85i1.1626

**Published:** 2018-10-09

**Authors:** Yolandi Rautenbach, Johan Schoeman, Amelia Goddard

**Affiliations:** 1 Department of Companion Animal Clinical Studies, University of Pretoria, South Africa

## Abstract

Canine babesiosis and ehrlichiosis are important tick-borne infections in South Africa. Many South African general veterinary practitioners perceive co-infection with *Ehrlichia* spp. as a common occurrence in dogs with babesiosis. Studies about the prevalence of co-infection in South African dogs are lacking. This retrospective study aimed to determine the prevalence of *Ehrlichia* co-infection in dogs with babesiosis. Additionally, the predicative value of specific haematological variables for co-infection was evaluated. The study population consisted of 205 dogs diagnosed with canine babesiosis presented to the Onderstepoort Veterinary Academic Hospital (OVAH) in 2006 and between 2011 and 2013. The *Babesia*-infected dogs were grouped based on presence or absence of an *Ehrlichia* spp. co-infection. *Ehrlichia* spp. co-infection was confirmed using polymerase chain reaction. Positive and negative predictive values (PPVs and NPVs) of leukopenia or thrombocytopenia for co-infection were also calculated. The prevalence of *Babesia* spp. and *Ehrlichia* spp. co-infection in this cohort of dogs was 2%. In the babesiosis dogs, the PPV of leukopenia for co-infection with *Ehrlichia* spp. was 1.3%, and the NPV 97.4%. Similarly, the PPV and NPVs of thrombocytopenia for co-infection were 2.1% and 100%, respectively. Co-infection with *Ehrlichia* spp. was a rare occurrence in dogs with babesiosis presented to the OVAH. Normal leukocyte or platelet counts confidently ruled out the presence of concurrent ehrlichiosis in this cohort of dogs. However, the diagnosis of *Ehrlichia* co-infection based on the presence of thrombocytopenia or leukopenia would have been associated with false positive results in more than 97.4% of cases.

## Introduction

Canine babesiosis and ehrlichiosis are important tick-borne infections in South Africa, resulting in severe clinical disease (Collett 2000; Rautenbach, Boomker & De Villiers 1991; Van Heerden 1982). In South Africa, canine babesiosis is predominantly caused by the virulent *Babesia rossi* sp. (Matjila et al. 2008). Although several *Ehrlichia* spp. are able to cause natural disease in dogs, only *E. canis* and *E. ruminantium* occur in southern Africa (Kelly 2000; Neer et al. 2002).

Severe thrombocytopenia is a common finding in canine babesiosis (Kettner, Reyers & Miller 2003; Scheepers et al. 2011), as is leukopenia (Scheepers et al. 2011). Similarly in canine ehrlichiosis, thrombocytopenia and leukopenia are common haematological abnormalities in both the acute and chronic phase of the infection (Kelly 2000; Shipov et al. 2008). Despite the presence of severe thrombocytopenia, clinical bleeding is uncommon in canine babesiosis because of marked platelet activation and marked hyperfibrinogenaemia (Goddard et al. 2015b; Liebenberg et al. 2013); however, excessive bleeding is seen in cases with concomitant *Ehrlichia* spp. infections (Van Heerden, Reyers & Stewart 1983), most likely secondary to the thrombocytopenia and thrombocytopathia reported in this disease (Neer 1998).

In endemic areas, infection with multiple tick-borne pathogens is possible in individual animals, especially secondary to a heavy tick infestation (Shaw et al. 2001). A single tick species can act as a vector for multiple pathogens and simultaneous infection with different organisms is possible (Schouls et al. 1999; Shaw et al. 2001). In South Africa, a 19% infection rate of *E. canis* was noted in *Rhipicephalus sanguineus* ticks (Mtshali et al. 2017), but to the authors’ knowledge no reports are available on the infection rate of *B. rossi* in *Haemaphysalis elliptica* ticks.

Studies about the prevalence of *Babesia* and *Ehrlichia* co-infections in dogs in South African are lacking. The majority of veterinary practitioners in South Africa have reported diagnosing canine ehrlichiosis in their practices and more than half consider it an occasional to common co-infection in dogs with babesiosis (Collett 2000). Approximately one-quarter of these practitioners based their diagnosis of concurrent *Ehrlichia* spp. infection on the presence of normal white cell count or leukopenia, while half of the clinicians based their diagnosis on the presence of a thrombocytopenia (Collett 2000).

In addition, a considerable proportion of practitioners are inclined to treat for *Ehrlichia* spp. co-infection in dogs with babesiosis, without the former being definitively confirmed (Collett 2000). Tetracycline, chloramphenicol, imidocarb dipropionate and amicarbalide are effective in the treatment of ehrlichiosis (Neer 1998). However, doxycycline is considered the drug of choice and a 4-week oral course is recommended based on the current consensus statement of the Infectious Disease Study Group of the American College of Veterinary Internal Medicine (Eddlestone et al. 2007; Neer et al. 2002). Currently, there are conflicting results about the efficacy of doxycycline in clearing *E. canis* from the blood and tissues of infected dogs (Eddlestone et al. 2007; Harrus et al. 1998; Iqbal & Rikihisa 1994; McClure et al. 2010), raising questions about the dosage and duration of doxycycline treatment and the development of potential drug resistance.

The first objective of this study was to determine the prevalence of *Ehrlichia* spp. co-infection in dogs with babesiosis presented to the Onderstepoort Veterinary Academic Hospital (OVAH). The second objective was to determine the predictive value of thrombocytopenia or leukopenia for co-infection with *Ehrlichia* spp. in dogs with babesiosis. We hypothesised that the prevalence of co-infection would be low in dogs presented with babesiosis and, additionally, that the presence of leukopenia or thrombocytopenia would not be predictive of concurrent *Ehrlichia* infection.

## Materials and methods

This retrospective study included the review of medical records of client-owned dogs naturally infected with canine *Babesia* spp., from two previously performed prospective clinical research studies. The dogs were presented for veterinary care to the OVAH, Gauteng, South Africa, between January and March 2006 (Study 1) and from October 2011 to April 2013 (Study 2).

The research protocols were approved by the University of Pretoria’s Animal Ethics Committee (V070-05 and V055-11, respectively). Several studies originating from these two cohorts of dogs have been published (Goddard et al. 2015a, 2015b, 2016; Rees & Schoeman 2008; Schoeman & Herrtage 2007, 2008).

Infection with *Babesia* parasites was initially diagnosed by demonstration of intra-erythrocytic trophozoites on stained thin blood smears, and the species was confirmed as *B. rossi* by polymerase chain reaction (PCR) and reverse line blot (RLB) (Matjila et al. 2004, 2008). All samples were also screened for *B. vogeli* and *E. canis*. Owner consent was obtained for enrolment of all the dogs in this study.

### Animals

Suitable dogs sampled during the second study were of any breed and either sex, > 12 weeks of age and weighed > 3 kg, while dogs from the first study did not have weight or age restrictions. Both groups of dogs had a demonstrable parasitaemia on a stained thin peripheral blood smear and clinical signs consistent with clinical babesiosis. Dogs were excluded if any signs of concurrent chronic or inflammatory disease conditions, any obvious infections or wounds, or any signs of trauma were present. Vaccination, drug therapy (drugs affecting platelet function or serum glucose concentrations, calcium-containing preparations, catecholamines or other adrenergic drugs, insulin therapy) or any unrelated metabolic illness four weeks prior to presentation were also reasons for exclusion.

### Sample collection and analyses

Peripheral venous blood was collected at presentation prior to any treatment. Blood samples were collected from the jugular vein from each dog with a 21-gauge needle by careful venepuncture with minimal stasis. The blood samples were collected into serum and ethylenediaminetetraacetic acid (EDTA) Vacutainer plastic tubes (Becton Dickinson [BD] Biosciences, New Jersey). The EDTA sample was used to perform a complete blood count (CBC), PCR and RLB assays. Because of the prolonged period between the two study populations, CBC data from two different automated cell counters were available: ADVIA 2120 (Siemens, Munich, Germany) and Cell-Dyn 3700 (Abbott Diagnostics, Santa Clara, CA, United States).

### Deoxyribonucleic acid extraction and polymerase chain reaction

Deoxyribonucleic acid (DNA) was extracted from 200 *μ*L EDTA-anticoagulated whole blood using a blood and tissue extraction kit (QIAmp blood and tissue extraction kit, Qiagen, Venlo, Netherlands) according to the manufacturer’s instructions. Molecular diagnosis of *B. rossi* and exclusion of other *Babesia*, *Theileria*, *Ehrlichia* and *Anaplasma* spp. were performed using PCR and RLB. Polymerase chain reaction was conducted with a set of primers that amplified a 460–540 base pair fragment of the 18S SSU rRNA spanning the V4 region, a region conserved for *Babesia* and *Theileria*. The *Ehrlichia* PCR amplified the V1 hypervariable region of the 16S SSU rRNA. The membrane used for RLB included probes for *B. vogeli*, *B. rossi*, *B. canis* and *E. canis* (Matjila et al. 2008).

### Statistical analysis

The *Babesia*-infected dogs were divided into groups, based on presence or absence of *Ehrlichia* spp. co-infection. Descriptive statistics were performed using a commercial software package (SPSS Statistics 23.0^®^ Software; SPSS Inc, StataCorp, College Station, TX, United States). The prevalence of *Ehrlichia* spp. co-infection in babesiosis dogs was defined as the percentage of babesiosis dogs that tested positive on PCR for *Ehrlichia* spp. The respective positive predictive values (PPVs) of leukopenia and thrombocytopenia for co-infection with *Ehrlichia* spp. were calculated. PPV = TP / (TP + FP) × 100, where TP represents the true positives (*Babesia*-infected dogs with leukopenia or thrombocytopenia co-infected with *Ehrlichia* spp.) and FP represents the false positives (*Babesia*-infected dogs with leukopenia or thrombocytopenia without *Ehrlichia* spp. co-infection). The respective negative predictive values (NPVs) of a normal leukocyte count and platelet count for the exclusion of co-infection with *Ehrlichia* spp. were also determined. NPV = TN / (TN + FN) × 100, where TN represents the true negatives (*Babesia*-infected dogs with normal leukocyte counts or platelet counts without *Ehrlichia* spp. co-infection) and FN represents false negatives (*Babesia*-infected dogs with normal leukocyte counts or platelet counts with *Ehrlichia* spp. co-infection). Thrombocytopenia was defined as a platelet count < 200 × 10^9^/L (population-based reference interval [RI]: 200–500 × 10^9^/L) and a leukopenia was defined as a leukocyte count < 6 × 10^9^/L (RI: 6–15 × 10^9^/L).

## Results

### Study population characteristics

Of the 205 dogs sampled that were naturally infected with *Babesia* spp., only 198 dogs were included in the study. Two dogs were excluded because of negative PCR results for any infectious agents and six dogs did not have any PCR results available for analysis. The median age (range) and weight (range) of all the infected dogs were 18 months (9–36) and 18.2 kg (8.5–28.5) (Table 1), respectively, and included 127 male and 71 female dogs. The most prevalent breeds were mixed-breed dogs (29%), followed by Boerboels (12%) and Jack Russell terriers (8%). The remaining breeds each represented less than 5% of the population. The median age (range) and weight (range) of the *Babesia*-infected dogs were 18 months (9.0–36.5) and 18.3 kg (8.6–28.5), respectively. The median age (range) and weight (range) of the co-infected dogs were 12 months (6–18) and 6.7 kg (4.2–23.9), respectively (Table 1).

**TABLE 1:** Population characteristics, leukocyte count and platelet count for dogs with *Babesia* spp. and *Ehrlichia* spp. infection.

Variable	All infected dogs			*Babesia*-infected only			*Babesia* spp. and *Ehrlichia* spp. co-infection		
	Mean ± SD	Median	IQR	Mean ± SD	Median	IQR	Mean ± SD	Median	IQR
Age (months)	30.67 ± 31.83	18.00	9.00–36.00	30.96 ± 32.00	18.00	9.00–36.50	12.00 ± 6.00	12.00	6.00–18.00
Weight (kg)	19.88 ± 13.49	18.20	8.50–28.50	20.10 ± 13.50	18.30	8.60–28.50	11.60 ± 11.77	6.70	4.20–23.90
Leukocyte count (×10^9^/L)	9.28 ± 6.69	6.70	4.80–11.90	9.30 ± 6.74	6.67	4.80–11.90	7.98 ± 3.92	8.86	3.88–11.21
Platelet count (×10^9^/L)	36 ± 62	17.00	4.00–49.00	35.00 ± 62.00	16.00	4.00–48.00	63.00 ± 37.00	58.00	31.00–100.00

SD, standard deviation; IQR, interquartile range

### Co-infection prevalence

Based on PCR results, 191 of the dogs were solely infected with *B. rossi* (96%), four dogs were infected with *B. vogeli* (2%), of which two were co-infected with *E. canis* (1%), one dog was co-infected with both *B. rossi and B. vogeli* (0.5%) and two dogs infected with *B. rossi* were co-infected with *E. canis* and *E. ruminantium* (1%). The prevalence of *Babesia* spp. and *Ehrlichia* spp. co-infection in this cohort of dogs was 2%.

### Predictive value of haematology results

The median leukocyte count (range) and platelet count (range) of all the infected dogs were 6.7 × 10^9^/L (4.8–11.90) and 17 × 10^9^/L (4–49), respectively. In the *Babesia*-infected dogs, the median leukocyte count (range) was 6.67 × 10^9^/L (4.8–11.90) and the median platelet count (range) was 16 × 10^9^/L (4–48). The median leukocyte count (range) in co-infected dogs was 8.86 × 10^9^/L (3.88–11.21), and the platelet count (range) was 58 × 10^9^/L (31–100) (Table 1).

The PPV of leukopenia for co-infection with *Ehrlichia* spp. in babesiosis dogs was 1.3%, while the NPV was 97.4% (Table 2). The PPV and NPV of thrombocytopenia for co-infection with *Ehrlichia* spp. in babesiosis dogs was 2.1% and 100%, respectively (Table 3).

**TABLE 2:** Contingency table illustrating the relationship between infection status and leukocyte count.

Variable	Infection status				Total	
	*Babesia* spp. and *Ehrlichia* spp. co-infection		*Babesia*-infected only			
	Number	%	Number	%	Number	%
Leukopenia	1	1.3	78	98.7	79	100.0
Normal leukocyte count	3	2.6	113	97.4	116	100.0
**Total**	**4**	**2.1**	**191**	**97.9**	**195**	**100.0**

**TABLE 3:** Contingency table illustrating the relationship between infection status and platelet count.

Variable	*Infection status*				Total	
	*Babesia* spp. and *Ehrlichia* spp. co-infection		*Babesia*-infected only	
	Number	%	Number	%	Number	%
Thrombocytopenia	4	2.1	189	97.9	193	100.0
Normal platelet count	0	0.0	2	100.0	2	100.0
**Total**	**4**	**2.1**	**191**	**97.9**	**195**	**100.0**

## Discussion

Published data on the prevalence of co-infection with multiple tick-borne pathogens in South African dogs are very limited. Our study showed that the prevalence of concurrent infections with *Babesia* and *Ehrlichia* spp. in South African dogs is very low. Our results concur with those of a previous molecular survey conducted on 1138 blood specimens collected from domestic dogs in South Africa between 2000 and 2006, which reported a 2% prevalence of *B. rossi* and *E. canis* co-infection (Matjila et al. 2008). Interestingly, the survey reported that *B. rossi* and *E. canis* co-infections were found in all of the sampled areas except for the Free State and Eastern Cape provinces (Matjila et al. 2008). Similar to our findings, 1.5% of dogs were concurrently infected with *B. rossi* and *E. canis*, 1.3% of the dogs had a *B. vogeli* and *E. canis* co-infection and only one dog had a concurrent *B. rossi* and *B. vogeli* infection in the subset of survey samples collected from the OVAH (Matjila et al. 2008).

Our results confirmed that *B. rossi* is the most common cause of babesiosis in dogs presented to the OVAH (Matjila et al. 2008). This correlates with the high percentage of *Babesia*-infected dogs presented to the OVAH that are infested with *H. elliptica*, the tick vector for *B. rossi* (Horak 1995; Matjila et al. 2008). The *B. vogeli* and *E. canis* co-infection can be explained by the fact that *R. sanguineus* is the vector for both of these pathogens (Groves et al. 1975; Uilenberg et al. 1989). The mixed infections with *B. rossi* and *E. canis* or *B. vogeli* is most likely because of the overlapping distribution pattern of *R. sanguineus* and *H. elliptica* and the fact that these vectors commonly infest the same host (Horak 1995; Matjila et al. 2008).

Two of the dogs in our study were co-infected with both *E. canis* and *E. ruminantium*. *Ehrlichia ruminantium* causes heartwater or cowdriosis in all domestic and some wild ruminants and is transmitted by ticks of the genus *Amblyomma* (Allsopp 2010). The main vector of heartwater in southern Africa is *A. hebraeum* (Allsopp 2010). Currently, eight different srRNA genotypes of *E. ruminantium* are known, of which only the Pretoria North genotype has been identified in dogs (Allsopp 2010). This genotype was isolated in South African dogs that presented with clinical symptoms of ehrlichiosis but that tested negative using a PCR assay specific for North American *E. canis* (Allsopp & Allsopp 2001). The *E. ruminantium* organism infecting dogs has not yet been isolated in culture (Allsopp 2010; Allsopp & Allsopp 2001), thus prohibiting further investigation into its pathogenicity, host specificity and the identification of its vector. Recently, a low number of *A. hebraeum* ticks were found in a South African cohort of dogs and cats (Mtshali et al. 2017). These ticks were negative for the presence of *E. canis* DNA (Mtshali et al. 2017), but the samples were not screened for *E. ruminantium* DNA.

Positive predictive value and NPV are utilised for assessing the probability of an accurate diagnosis (Petrie & Watson 2013). There is an interdependence between prevalence, sensitivity, specificity and predictive values (Hernaez & Thrift 2017). Prevalence can also be interpreted as the probability that the disease is present before the test is performed (pretest probability) and affects predictive values (Petrie & Watson 2013). When disease prevalence is low, the NPV is higher and consequently the PPV lower (Hernaez & Thrift 2017; Petrie & Watson 2013). Thus, in a low prevalence setting, a negative result confidently excludes the presence of disease (Hernaez & Thrift 2017; Petrie & Watson 2013). The opposite is true when the prevalence of the disease increases (Petrie & Watson 2013). The predictive value of the presence of leukopenia or thrombocytopenia for concurrent ehrlichiosis in our population of dogs with babesiosis was very low, 1.3% and 2.1%, respectively. In contrast, the predictive value of normal leukocyte count or platelet count for the absence of concurrent ehrlichiosis was very high, 97.4% and 100%, respectively. Therefore, in light of the low prevalence of co-infection in this population of dogs with babesiosis, a negative test result (i.e. normal leukocyte count or normal platelet count) confidently rules out the presence of concurrent ehrlichiosis. Furthermore, a positive test result (i.e. leukopenia or thrombocytopenia) may result in incorrect diagnosis of co-infection, leading to an over-diagnosis of concurrent ehrlichiosis in *Babesia*-infected dogs.

The median age of the population of infected dogs in this study was 18 months, which is consistent with previous reports that the majority of dogs with babesiosis are young (Jacobson 2006; Mellanby et al. 2011). More male than female dogs were presented with babesiosis, 64% compared to 36%, mirroring previous reports (Jacobson 2006). The most common breeds in this study were mixed-breed, followed by Boerboel and Jack Russell terrier. These findings are similar to the reported literature, with mixed-breed dogs most commonly presented for babesiosis while toy breed dogs appear to be less likely to be presented for babesiosis (Jacobson 2006; Mellanby et al. 2011).

A limitation of this study is the fact that the haematology was performed on two different haematology analysers and therefore the results are not comparable. However, the population-based RIs remained unchanged over the study period; therefore, the classification of a leukopenia or thrombocytopenia remained consistent. A further limitation regarding the reported prevalence of *Ehrlichia* co-infection in South African dogs with babesiosis is that the findings of this study are only applicable to the geographical area of this cohort of dogs. The prevalence might be higher in areas where regular tick prevention is not practised or in areas with a higher concentration of tick vectors. In light of the retrospective nature of this study, the inference was made that the analysed data were unbiased; however, some babesiosis cases with concurrent ehrlichiosis could have inadvertently been excluded. Therefore, the results reported here should be confirmed with a prospective study.

## Conclusion

It can thus be concluded from these results that concurrent ehrlichiosis is uncommon in dogs with babesiosis presented to the OVAH, situated north of Pretoria in Gauteng. Although thrombocytopenia is a common finding in dogs with babesiosis, its PPV for co-infection is very low, similar to the presence of leukopenia. Therefore, the diagnosis of *Ehrlichia* co-infection based on thrombocytopenia or leukopenia is associated with many FP results.
